# The Influence of Ti and Al on the Evolution of Microstructure and Mechanical Properties in Medium-Entropy and High-Entropy Alloys Based on Al_x_Ti_x_CrFe_2_Ni_2_

**DOI:** 10.3390/ma18061382

**Published:** 2025-03-20

**Authors:** Róbert Kočiško, Patrik Petroušek, Karel Saksl, Ivan Petryshynets, Ondrej Milkovič, Dávid Csík

**Affiliations:** 1 Faculty of Materials, Metallurgy and Recycling, Technical University of Košice, Letna 9, 04200 Kosice, Slovakia; patrik.petrousek@tuke.sk (P.P.); karel.saksl@tuke.sk (K.S.); 2 Institute of Materials Research, Slovak Academy of Sciences, Watsonova 47, 04001 Košice, Slovakia; ipetryshynets@saske.sk (I.P.); omilkovic@saske.sk (O.M.); dcsik@saske.sk (D.C.); 3 Institute of Experimental Physics, Slovak Academy of Sciences, Watsonova 47, 04001 Košice, Slovakia

**Keywords:** medium-entropy alloys, high-entropy alloys, microstructural evolution, mechanical properties, arc melting

## Abstract

This study focuses on the cobalt-free medium-to-high-entropy alloys Al_x_CrFe_2_Ni_2_ and Al_x_Ti_x_CrFe_2_Ni_2_, investigating the influence of Al_x_ and Ti_x_ (where x = 0.2, 0.3, 0.4, 0.5, and 0.6) on the development of microstructural and mechanical properties in as-cast and annealed states. Structural changes were examined using optical microscopy, scanning electron microscopy (SEM), and X-ray diffraction (XRD) measurements, while mechanical properties were evaluated through Vickers hardness testing and compression testing. X-ray diffraction analysis of the Al_x_CrFe_2_Ni_2_ alloys confirmed that increasing the Al content in the as-cast state leads to the formation of a BCC phase, which completely dissolves into the FCC matrix after homogenization annealing. These single-phase alloys exhibit good ductility with relatively high strain hardening, such as the Al_0.6_CrFe_2_Ni_2_ alloy, which achieved a maximum compressive strength of $\sigma_{max}=1511\;MPa$ at 50% deformation. A significant strengthening effect of Ti was observed in the Al_x_Ti_x_CrFe_2_Ni_2_ alloys, the mechanical properties of which are closely linked to the higher BCC phase content in the homogenized structure. The highest compressive strength, $\sigma_{max}=2239\;MPa$, was achieved by the Al_0.5_Ti_0.5_CrFe_2_Ni_2_ alloy, which fractured via a transcrystalline brittle fracture at 43% deformation. All alloys investigated offer an excellent balance between strength and ductility, which could meet the requirements of demanding structural applications.

## 1. Introduction

Materials research in the field of medium entropy alloys (MEAs) and high-entropy alloys (HEAs) offers virtually unlimited possibilities for investigation, which is increasingly gaining significant attention and appeal, mainly due to their interesting mechanical and physical properties, such as high strength [1,2,3], excellent ductility [4], good thermal stability [5], wear resistance [6], corrosion resistance [7,8], high radiation tolerance [9], good electrochemical performance [10,11,12] and hydrogen affinity [13], etc. This is a relatively new type of alloy that, thanks to the high entropy of mixing, forms simple solid solutions such as face-centered cubic (FCC), body-centered cubic (BCC), or hexagonal close-packed (HCP) structures. In this way, they suppress the formation of intermetallic compounds and other complex phases, which is significantly different from traditional alloys based mainly on one or two constituent elements [14,15,16].

Current research focuses on the modification of single-phase HEAs/MEAs by adding additional alloying elements to improve their mechanical properties through the exclusion of one or more strengthening phases. A typical representative of these alloys is the Al-modified alloy Al_x_CoCrFeNi [17], which offers a wide range of microstructures and mechanical properties at different Al atomic concentrations. A further increase in strength can also be achieved by adding Ti, where the AlCoCrFeNiTi_0.5_ alloy [18] exhibits excellent mechanical properties in compression, where the yield strength is $\sigma_{Y}=2260\;MPa$, the compressive strength $\sigma_{f}=3140\;MPa$, and the ductility $\varepsilon_{f}=23\%$. In addition, various conventional strategies of thermo-mechanical strengthening have been used for the additional strengthening of these structures, including precipitation, grain boundaries, work hardening, twinning-induced plasticity, transformation-induced plasticity, etc. Most HEAs/MEAs contain cobalt in their chemical composition, which gives the alloys good mechanical properties, but their high price makes the use of these alloys in commercial structural applications ineffective because they cannot compete with today’s advanced steel. This stimulated the development of so-called Co-free HEAs/MEAs, which created space for the creation of new chemical concepts. Several strategies have been proposed, such as systems in which the stabilizing effect of Co on the FCC phase is replaced by another element such as Cu: AlCrCuFeNi_2_, AlCrCuFeNi [19,20]), Mn: CuFeMnNi [21], and others. Another approach is to replace Co with a higher proportion of one of the basic elements, such as Ni: Al_x_CuCrFeNi_2_, Ni_2_CrFeAl_0.3_Ti_x_, Al_x_CrFe_2_Ni_2_, AlCrCuFeNi_3_ [22,23,24,25,26]; Fe: Al_0.3_CrFe_1.5_MnNi_0.5_Ti_x_, Fe_40.4_Ni_11.3_Mn_34.8_Al_7.5_Cr_6_ [27,28,29]; and others. From the survey of Co-free alloys, it can be clearly seen that there is a great effort to find optimal compositions and satisfy the required mechanical properties, mainly in non-equimolar multi-element MEA/HEA compositions.

This study focuses on Co-free medium-to-high-entropy alloys, the core alloy of which is a non-equimolar CrFe_2_Ni_2_ system. It systematically investigates the influence of Al and Ti of Al_x_CrFe_2_Ni_2_ and Al_x_Ti_x_CrFe_2_Ni_2_ alloys (where x = 0.2, 0.3, 0.4, 0.5, and 0.6) on phase evolution, crystal structure, hardness, and compressive mechanical properties in the as-cast and homogenized states.

## 2. Materials and Methods

Two series of alloys were used for the experiment: one series used medium-entropy MEA with nominal compositions of Al_x_CrFe_2_Ni_2_ (where x represents the molar ratio and x = 0.2, 0.3, 0.4, 0.5, 0.6, and thus the alloys are denoted as Al2, Al3, Al4, Al5, and Al6, respectively) and the second series used medium-to-high-entropy alloys and high-entropy HEA with nominal compositions of Al_x_Ti_x_CrFe_2_Ni_2_ (where x represents the molar ratio and x = 0.2, 0.3, 0.4, 0.5, 0.6, and thus the alloys are denoted as Al2Ti2, Al3Ti3, Al4Ti4, Al5Ti5, and Al6Ti6, respectively). All alloys were prepared in the form of small ingots produced by the arc melting method in a vacuum-arc-melting furnace (Mini Arc Melter MAM-1). The melting process was carried out in an argon atmosphere on a water-cooled copper substrate. To ensure chemical homogeneity, each ingot was remelted 5 times while the ingots were flipped after each melting process. The raw materials Fe, Ni, Cr, Al, and Ti had a purity higher than 99.9 wt. %. The cast structures were subsequently homogenized at 1150 °C for 5 h.

Diffraction measurements were carried out by a Rigaku Rapid II (Rigaku Holdings Corporation, Tokio, Japan) diffractometer equipped with the 2D-curved detector D-max. The Mo lamp irradiated the sample with a reduced beam size to a 0.8 mm collimator hole. The reflection geometry was used by setting a fixed omega motor of 20 degrees and phi motor oscillation at 15 degrees/seconds. The following standard metallographic procedures were used to observe the microstructures: sectioning, preparation, grinding, polishing, and etching. To display the crystallographic structures, all alloys were etched in a solution of marble (HCl + CuSO_4_ + methanol) for 1–5 s. Low magnifications were observed on an optical microscope (OM) ZEISS AXIO VERT A1 (Carl Zeiss, Oberkochen, Germany), and details were given at high resolutions on a scanning electron microscope (SEM) JEOL JSM-7000F (JEOL, Tokyo, Japan) with the energy dispersive spectroscope (EDS, Oxford Inca X-sight model 7557). The chemical composition of the individual structural components was measured in more than three different areas. The phase maps of selected microstructures were obtained using electron back-scatter diffraction (EBSD), Oxford Nordlys Max^2^, (Oxford Instruments, UK, on a TESCAN MIRA 3 LMU, Brno, Czech Republic). The volume fraction of each phase in the alloys was estimated by using digital image analysis in more than five different areas at different magnifications.

The mechanical properties were determined using the compression tests carried out at room temperature using the universal mechanical testing machine Tinius Olsen H300KU (Tinius Olsen, Horsham, USA). The compression tests of each alloy were performed on three specimens under the same conditions of strain rate of 1 × 10^−3^ s^−1^, with maximum deformation of the cylindrical specimen height of up to 50%. The alloys were tested after homogenization annealing on cylindrical samples (*Φ* 4 × 8 mm). The structural components of the alloys were measured by the microhardness on the Vickers hardness tester (Struers Duramin 5 (Struers, Kodane Denmark). The measurement was carried out on the etched surface of the samples, while each structural component was measured with at least 10 punctures at a load of 981 mN (HV0.1) for 5 s.

## 3. Results

### 3.1. Characterization of Structural and Mechanical Properties of Al_x_CrFe_2_Ni_2_ Alloys

**Figure 1:**
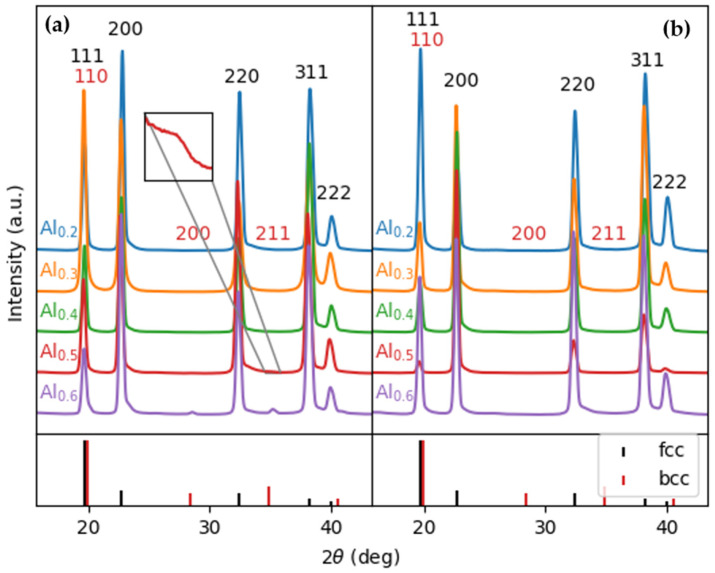
XRD patterns of the Al_x_CrFe_2_Ni_2_ alloys: (**a**) as cast and (**b**) as annealed.

The XRD patterns from the as-cast Al_x_CrFe_2_Ni_2_ alloys are displayed in Figure 1a. In alloys Al2, Al3, and Al4, only the FCC phase was identified, while in Al5 alloys, a small diffraction peak corresponding to (220) BCC (near 35°) began to emerge. With a higher Al content, this peak becomes more intense, as can be seen in the diffraction pattern of the Al6 alloy. In addition, two other peaks corresponding to (211) BCC (near 28°) and (110) BCC (near 20°) appeared in the Al6 alloy, as shown by the details of these patterns. This indicates that the addition of Al increases the volume fraction of the BCC phases at the expense of the FCC phase, which was also observed in [25]. Figure 1b shows the XRD patterns from Al_x_CrFe_2_Ni_2_ in the annealed state, where it is clearly seen that all alloys have a single-phase FCC structure. With the increasing Al content, a slight increase in the lattice constant can be observed in the FCC phase, which is presented in Table 1. This increase indicates the formation of a substitute solid solution, which is also reflected in a slight shift in the diffraction peaks to smaller angles.

**Figure 2:**
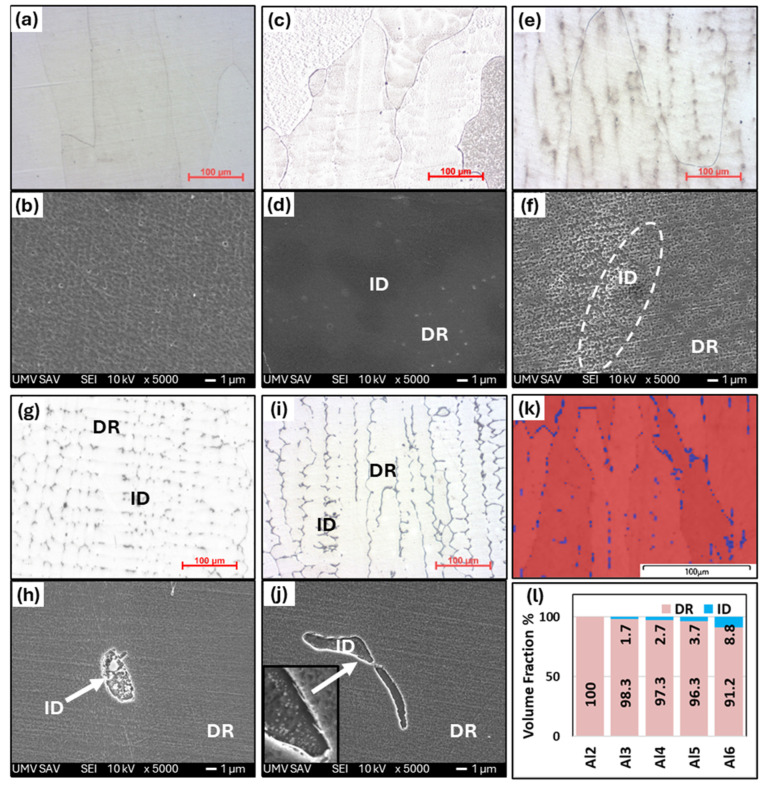
The microstructure of as-cast Al_x_CrFe_2_Ni_2_ alloys, with low-magnification images (500×) and high-magnification SEM images (5000×): Al2 (**a**,**b**), Al3 (**c**,**d**), Al4 (**e**,**f**), Al5 (**g**,**h**), Al6 (**i**,**j**), EBSD phase map of the Al6 alloy (**k**) and volume fraction of DR and ID phases (**l**).

**Figure 3:**
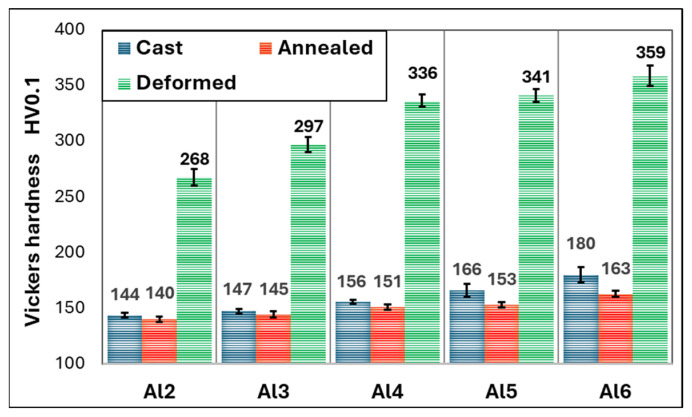
Microhardness of Al_x_CrFe_2_Ni_2_ alloys in cast, annealed, and deformed states.

Figure 2 shows the microstructures of as-cast Al_x_CrFe_2_Ni_2_ alloys at different magnifications, where low magnification is attained from an optical microscope and high magnification is attained from SEM. The chemical composition of the individual structural phases and regions is given in Table 2. The Al2 alloy exhibits a single solid solution structure that crystallizes into large columnar grains oriented in the direction of the solidification of the melt, as documented in Figure 2a,b. The Al3 and Al4 alloys have similar microstructures, while inside the columnar grains, there is a local mixing of elements, which are not yet bound by the interface from the matrix; it probably represents the dendritic nuclei that formed (Figure 2d,f). This phenomenon was also observed in [17]. The chemical composition of the DRs is close to the nominal composition of the designed alloys. The ID regions have an increased Al and Ni content and a reduced Cr and Fe content. Their volume fracture is up to 3%. However, their volume fraction was too small to be captured by the XRD measurement, which revealed only a single-phase structure, as shown in Figure 1a. The Al5 and Al6 alloys have a columnar dendritic structure, which is formed by a light matrix, the so-called dendritic region (DR), and a dark phase, which is clearly delimited from the matrix by an interface, the so-called inter-dendritic region (ID) shown in Figure 2h,j. It is evident that with the increasing Al content, the proportion of the ID region also increases [25,30,31], which for the Al5 and Al6 alloys reaches a volume fraction of 3.7% and 8.8%, respectively (Figure 2l). Based on XRD measurements, this is a BCC phase. The phase composition of the Al6 alloy is observed by EBSD in Figure 2k, where the FCC phase (red color) represents the DR and the BCC phase (blue color) represents the ID region. Phase identification was performed based on the lattice parameters obtained from XRD measurements. According to the literature [25], the ID region is composed of the BCC + B2 phase, while our measurements did not detect this. In Figure 3, the graphical dependence of Vickers hardness on alloys can be seen in the cast state, where hardness increases proportionally with the increasing Al content. The microhardness measurements also show that the Al2, Al3, and Al4 alloys have a homogeneous single-phase structure, as they show low standard deviations (σ_HV_ = ±2.5). In contrast, the Al5 and Al6 alloys show higher standard deviations (σ_HV_ = ±6), which is characteristic of the multiphase composition of these alloys.

**Figure 4:**
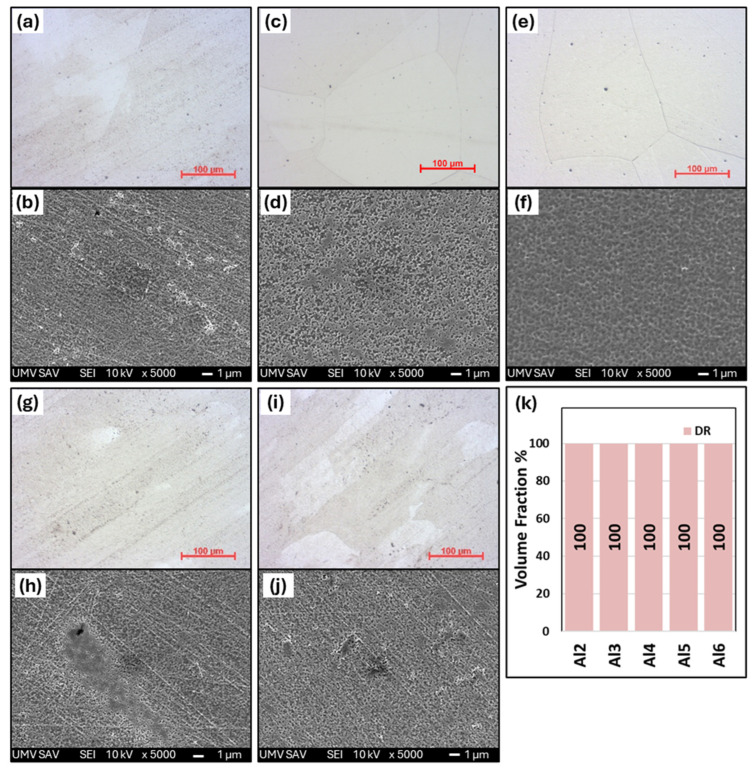
The microstructure of as-annealed Al_x_CrFe_2_Ni_2_ alloys with low-magnification images (500×) and high-magnification SEM images (5000×): Al2 (**a**,**b**), Al3 (**c**,**d**), Al4 (**e**,**f**), Al5 (**g**,**h**), Al6, (**i**,**j**), and (**k**) volume fraction of DR and ID phases.

Figure 4 shows the microstructural changes in Al_x_CrFe_2_Ni_2_ alloys after homogenization annealing. Homogenization annealing was carried out in order to homogenize the chemical composition in the entire volume and obtain a single-phase alloy with high plasticity. As we can see, all alloys have a coarse-grained polyhedral structure after annealing with a grain size of 200–500 μm. No precipitates of secondary phases were observed in the structures. Even alloys with a higher aluminum content, such as Al5 and Al6, in the ID region were completely dissolved into a solid solution (Figure 4g,i), which was confirmed by XRD analysis (Figure 1b). Due to sufficient annealing temperature and time, the chemical composition of all alloys was equalized (see Table 3), which confirms the correctly selected homogenization annealing parameters. The hardness of the annealed alloys is shown in Figure 3. The Al2, Al3, and Al4 alloys show only a slight decrease in hardness of up to 3%, while a more significant decrease is observed in the Al5 and Al6 alloys of up to 9%. All annealed states show a low standard deviation (σ_HV_ = ±2.5), which confirms the formation of a homogeneous single-phase structure.

**Figure 5:**
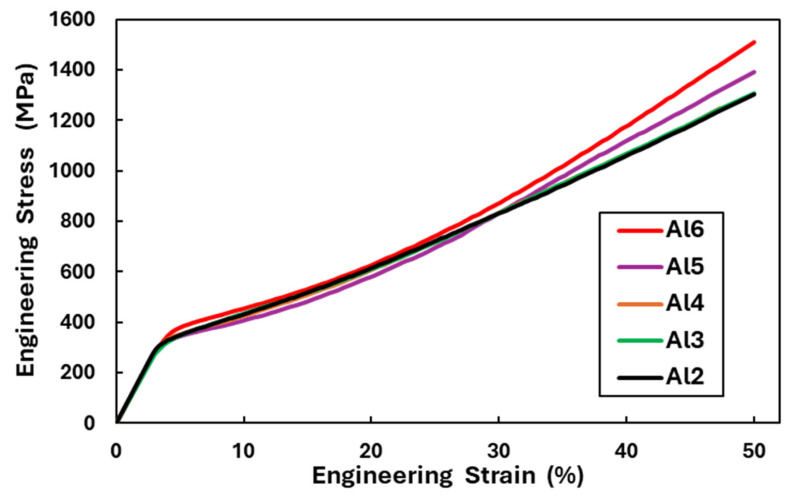
Compressive engineering stress–strain curves of the Al_x_CrFe_2_Ni_2_ alloys at room temperature.

The engineering stress–strain curves of the Al_x_CrFe_2_Ni_2_ alloys from the compressive test are shown in Figure 5. The mechanical properties of all the alloys are listed in Table 4. The graph (Figure 5) clearly shows that alloys have similar mechanical properties, and their deformation behavior is almost the same; even the course of alloys Al_2_, Al_3_, and Al_4_ completely overlap. Slight changes can be seen in yield stress $\sigma_{Y}$, as shown in Table 4. The Al_6_ alloy has the highest $\sigma_{Y}=327\;MPa$. From the measured values, it can be concluded that the aluminum content, ranging from 3.85 to 10.71 at. % (Al_2_–Al_6_), had only a slight effect on the $\sigma_{Y}$ value, where $∆\sigma_{Y}=57\;MPa$, if the alloys were in the as-annealed state after homogenization annealing (1150 °C for 5 h). In the case of maximal stress $\sigma_{max}$ at deformation ε=50% for the test cylinders, only slight changes were measured between the Al_2_, Al_3_, Al_4_, and Al_5_ alloys ($∆\sigma_{max}=70\;MPa$). In contrast, the Al_6_ alloy showed the highest rate of strengthening $\sigma_{max}=1511\;MPa$ among all the alloys, where the difference in values with the Al_2_ alloy was $∆\sigma_{max}=209\;MPa$. It should be noted that no surface damage was observed on any of the alloys after deformation =50%, which confirms that the alloys have high formability at room temperatures. Microhardness values were measured in the central part of the samples after the compression test; their values are shown in Figure 3 (green bars). The deformed structures of all alloys show a more than two-fold increase in hardness, which confirms that the Al_x_CrFe_2_Ni_2_ alloys have very good formability.

### 3.2. Characterization of Structural and Mechanical Properties of Al_x_Ti_x_CrFe_2_Ni_2_ Alloys

**Figure 6:**
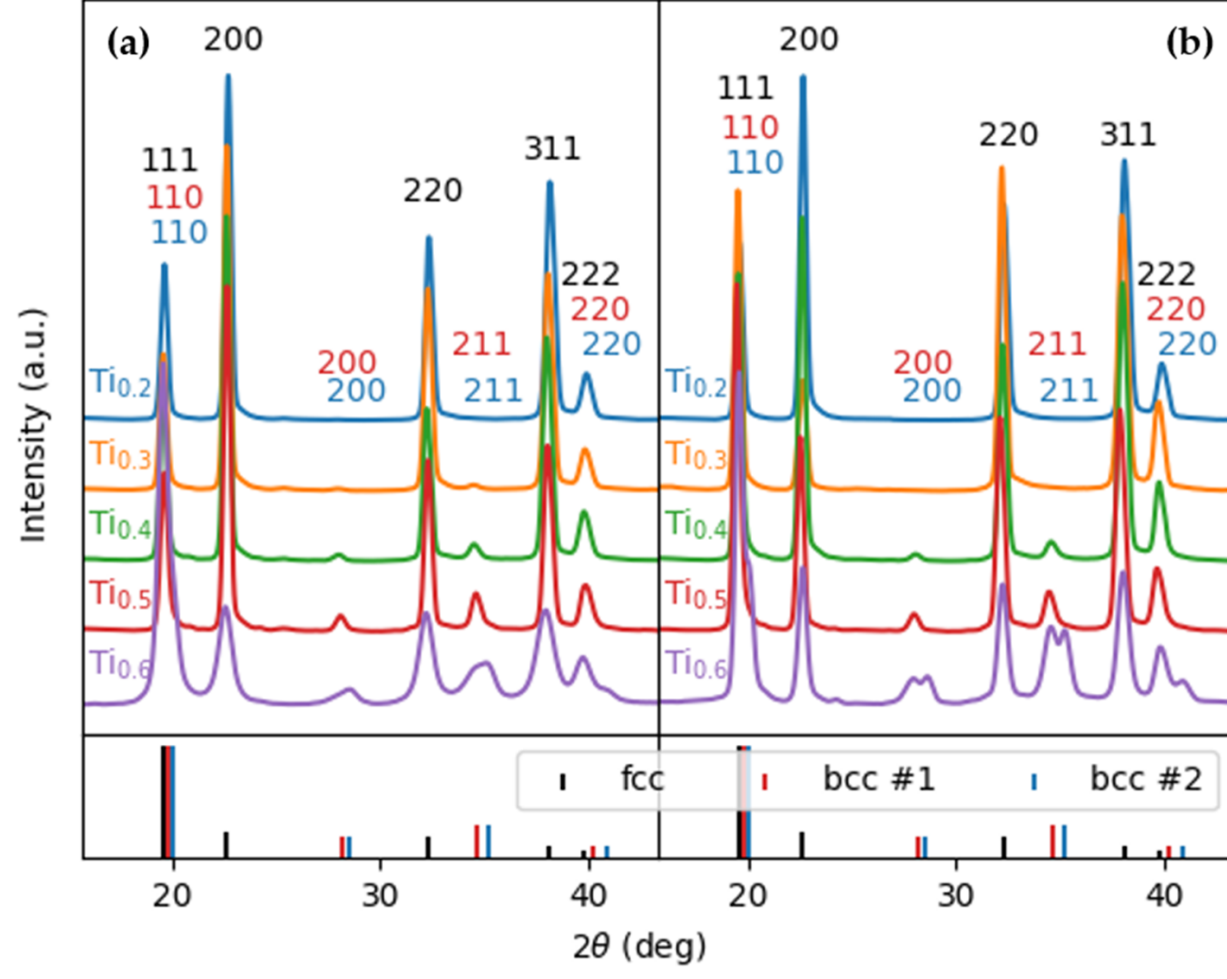
XRD patterns of the Al_x_Ti_x_CrFe_2_Ni_2_ alloys: (**a**) as cast and (**b**) as annealed.

The XRD patterns from the as-cast Al_x_Ti_x_CrFe_2_Ni_2_ alloys are displayed in Figure 6a. Only one alloy, Al2Ti2_,_ exhibited a single-phase structure in the as-cast state. The first signs of a BCC phase appeared in the Al3Ti3 alloy, where two small diffraction peaks corresponding to (220) BCC (near 35°) and (221) BCC (near 28°) were created. These two peaks intensified with the increasing Al and Ti content, which could be observed in the XRD patterns of the Al4Ti4 and Al5Ti5 alloys, where the BCC phase appeared as a secondary phase. For the Al6Ti6 alloy, two BCCs were present simultaneously, namely the BCC1 phase and the BCC2 phase, the peaks of which are very close to each other. Their diffraction peaks correspond to (110) near 20°, (200) near 28°, (211) near 35 and (220) near 42°. Their identification requires a more precise method, such as transmission electron microscopy (TEM).

The XRD patterns in the annealed Al_x_Ti_x_CrFe_2_Ni_2_ alloys are displayed in Figure 6b. Al2Ti2 and Al3Ti3 alloys after homogenization annealing showed a single-phase FCC structure, while in the Al4Ti4 and Al5Ti5 alloys, the presence of the BCC phase at the same 2θ positions was again confirmed. In the Al6Ti6 alloy, the structure recovered during annealing, which caused a narrowing of the peaks, and more clearly demonstrated the presence of two BCC phases than that observed in the as-cast state. The lattice parameters of the alloys in the as-cast and annealed states are given in Table 5. It can be observed that in the case of the BCC phases, the lattice parameter decreased, which was a result of a shift in diffraction peaks to higher angles. This phenomenon is typical of the formation of ordered B2 phases, such as NiAl or NiTi, where the regular alternation of atoms in the lattice leads to its compaction and subsequent change in the diffraction properties.

**Figure 7:**
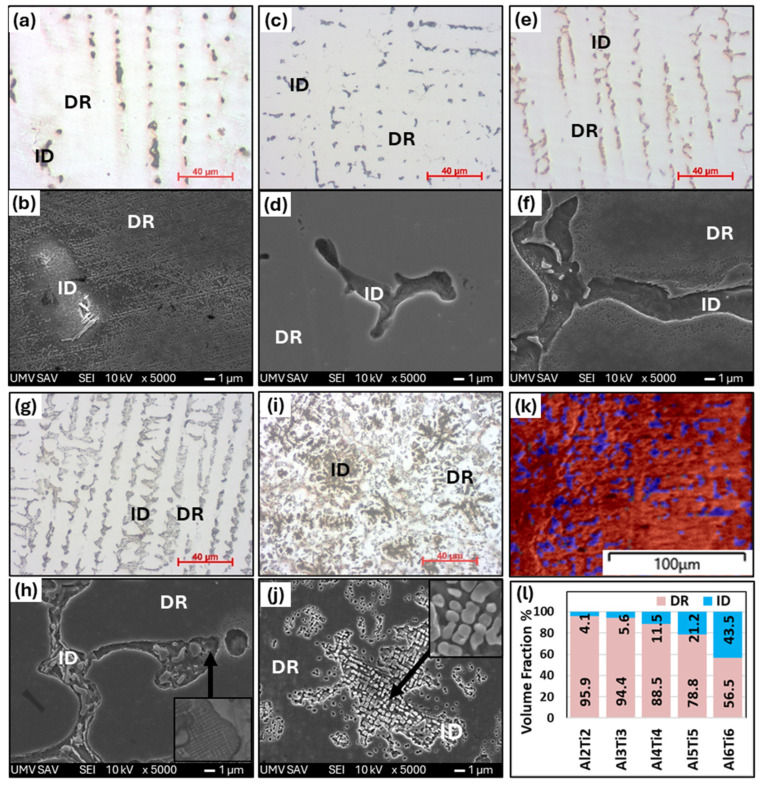
The microstructure of as-cast Al_x_Ti_x_CrFe_2_Ni_2_ alloys, with low-magnification images (500×) and high-magnification SEM images (5000×): Al2Ti2 (**a**,**b**), Al3Ti3 (**c**,**d**), Al4Ti4 (**e**,**f**), Al5Ti5 (**g**,**h**), Al6Ti6 (**i**,**j**), EBDS phase map of the Al5Ti5 alloy (**k**), and volume fraction of DR and ID phases (**l**).

**Figure 8:**
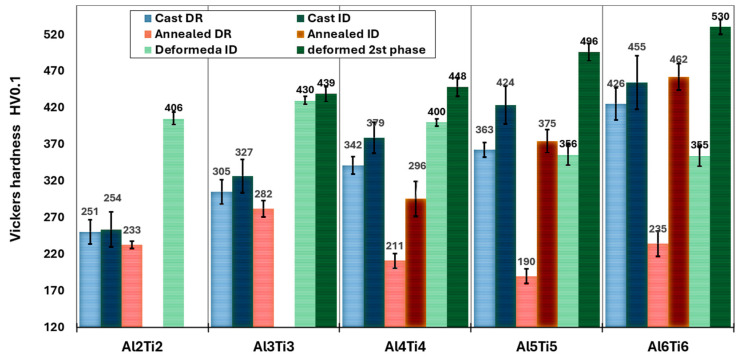
Vickers hardness of Al_x_Ti_x_CrFe_2_Ni_2_ alloys in as-cast and annealed states.

Figure 7 shows the effect of the Ti addition on the development of the microstructures of Al_x_CrFe_2_Ni_2_ alloys at different magnifications. In the Al2Ti2 alloy, unlike the alloy without Ti, it is possible to see the presence of a second phase arranged in a casting dendritic structure (DR and ID). According to the chemical composition, which is given in Table 6, it is possible to see that the ID phase was formed mainly by the presence of the Ti element since its content was 13.86%, while the presence of Al was suppressed to a content of 2.63%. Also, in this case, the volume fraction of the ID region was relatively low, 4.1% (Figure 7k); therefore, it was not captured by the XRD measurement (Figure 6a). With increasing equimolar proportions of Al and Ti in the Al3Ti3, Al4Ti4, and Al5Ti5 alloys, it was possible to observe that the ID regions increased uniformly, thereby increasing their volume fraction to 5.6, 11.5, and 21.2%, respectively. The alloys crystallized in a columnar dendrite structure, as shown in Figure 7c,e,g. This confirms that the addition of Ti intensively increased the precipitation of the second phase into ID regions, as observed in the Al-Ti-Co-Ni-Fe-based alloys [32,33]. From the chemical analysis (Table 6), it can be seen that the ID region in these alloys is relatively well stabilized because it shows the same ratio for all elements, namely a higher content of Al, Ti in a range from 18.2 to 19.8 at. % and Ni 44.7 at. %, and a lower content of Cr and Fe at the level of mean values ≈ 4.9; 12.7 at. %. The inter-dendrite region is formed by the matrix and thin stripes-like precipitates, as documented by the detail in Figure 7h. An inter-dendrite region with such a morphology was also identified in the alloys CrFeNiAl_3_Ti_3_ [34]**,** where it was identified as the BCC+ B2 phase. The presence of the BCC phase was also confirmed by the XRD measurement (as phase BCC 1, see Figure 6), while its distribution in the structure was shown using the phase map obtained from the EBSD measurement in Figure 7k. It was also seen that with the addition of Al and Ti, the volume fraction of the FCC phase decreased while the volume fraction of the BCC phase increased. The Al6Ti6 alloy has different structural characteristics, as it crystallizes in an equiaxed dendritic structure (Figure 7i), with a volume fraction of up to 43.5%. The interior of the ID region is globular-rod-shaped (Figure 7j), and is composed of two BCC phases according to XRD. The hardness of the structural components in the as-cast state of the Al_x_CrFe_2_Ni_2_ alloy is shown in Figure 8, where it can be seen that the hardness compared to alloys without a Ti content significantly increases in some cases by up to 57% (DR-Al6Ti6). From the measured values, it is evident that the ID region has a higher hardness than the DR, which confirms the presence of the BCC phase, which generally has higher mechanical properties. With the increasing Al and Ti content, there is a uniform strengthening of the DR and ID regions, where the highest hardnesses were achieved in the Al6Ti6 alloy: DR = 426 HV0.1 and ID = 455 HV0.1.

**Figure 9:**
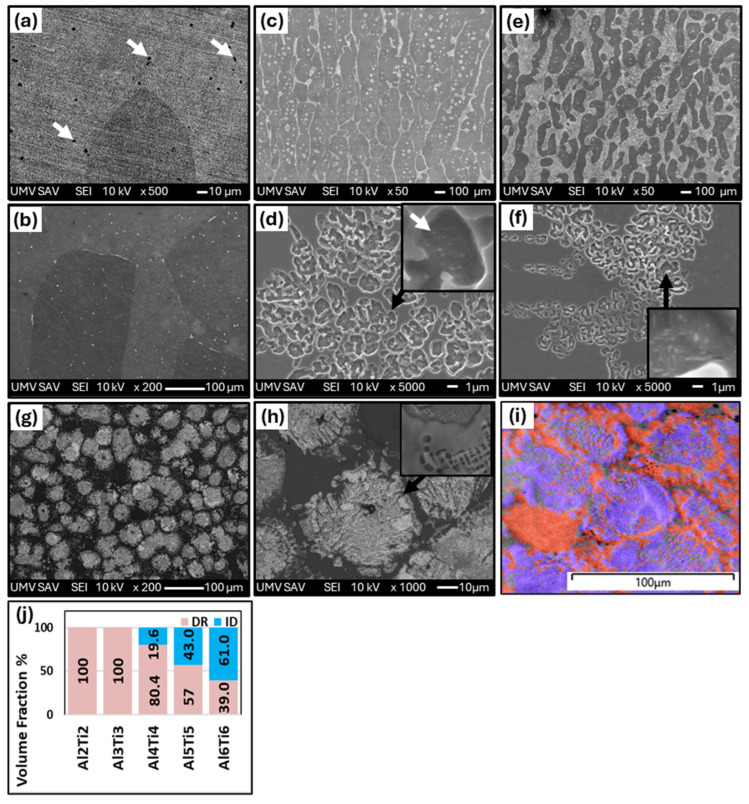
The microstructure of as-annealed Al_x_Ti_x_CrFe_2_Ni_2_ alloys. with low-magnification images (500×) and high-magnification SEM images (5000×): Al2Ti2 (**a**). Al3Ti3 (**b**). Al4Ti4 (**c**,**d**). Al5Ti5 (**e**,**f**). Al6Ti6 (**g**,**h**), EBSD phase map of the Al6Ti6 alloy (**i**), and volume fraction of DR and ID phases (**j**).

The microstructural changes in Al_x_Ti_x_CrFe_2_Ni_2_ alloys after homogenization annealing are shown in Figure 9. The structure of the Al2Ti2 and Al3Ti3 alloys after annealing is formed by large columnar grains in which the ID region is dissolved and the chemical composition is uniform (see Table 7); these results are also in full agreement with our XRD measurements. Several Ti precipitates (white arrows in Figure 9a) can be observed in the Al2Ti2 alloy, the size of which is approximately 5 μm. The Al4Ti4 alloy consists of a matrix (grain interior) and a phase preferentially excluded along the grain boundaries; part of this phase is also found inside the grains in the form of flakes (Figure 7b,c). Based on XRD measurements, a BCC phase is formed. A similar morphology of the structure appeared in Ni_2_CrFeAl_0.3_Ti_0.3_ alloys [24]. In the substructure of the Al4Ti4 alloy, several cuboidal particles with a size of about 200 nm were found in the BCC phase (white arrows at high magnification in Figure 9d). Particles with the same morphology in the ID phase were identified as the B2 phase in a similar Al_0.4_Ti_0.2_CrFeNi [34] alloy. In the Al5Ti5 and Al6Ti6 alloys, a two- to three-fold increase in the volume fraction of the BCC phase was observed at 43% and 61%, respectively. In the case of the Al6Ti6 alloy, an equiaxed dendritic structure was spheroidized during annealing. Spherical particles were formed by areas with fine stripe-like precipitates and areas with globular-rod-shaped precipitates, as documented in detail in Figure 9h. Each of these regions is formed by a different BCC phase, and their presence was also confirmed by EDX measurements. Figure 9i shows an EBSD map, which demonstrates that the spherical particles are composed of the BCC phase, while the regions in between are composed of the FCC phase. However, the EBSD measurement could not reliably distinguish the individual phases (BCC 1, BCC 2), probably due to the too-fine stripe-like precipitates, the thickness of which is less than 100 nm.

The change in hardness of the annealed Al_x_Ti_x_CrFe_2_Ni_2_ alloys is shown in Figure 8. A slight decrease in hardness occurred in the Al2Ti2 and Al3Ti3 alloys, where the hardness of the single-phase alloy was 233 HV0.1 and 282 HV0.1, respectively, which is comparable to the alloy Al_5_Ti_5_Co_35_Ni_35_Fe_20_ [32]. A marked decrease in hardness in the DR occurred for the Al4Ti4 and Al5Ti5 alloys, where the hardness was 211 HV0.1 and 190 HV0.1, respectively. The Al6Ti6 alloy had a relatively high hardness even after annealing, where DR had 235 HV0.1, and ID had 462 HV0.1.

**Figure 10:**
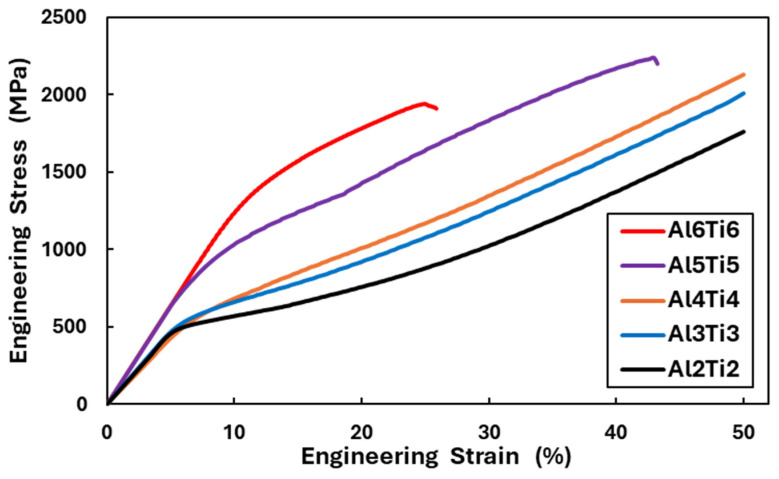
Compressive engineering stress–strain curves of the Al_x_Ti_x_CrFe_2_Ni_2_ alloys at room temperature.

The engineering stress–strain curves of the Al_x_Ti_x_CrFe_2_Ni_2_ alloys from the compressive test are shown in Figure 10. The mechanical properties of all the alloys are listed in Table 8. Compared to Al_x_CrFe_2_Ni_2_ alloys, it can be seen that Ti significantly increased the mechanical properties, whereas in the Al2Ti2, Al3Ti3, and Al4Ti4 alloys, the yield stress $\sigma_{Y}$ increased by ${∆\sigma }_{Y}\approx 200\;MPa$ ($\sigma_{Y,\;Al2Ti2}=447\;MPa$, $\sigma_{Y,\;Al3Ti3}=475\;MPa$ and $\sigma_{Y,\;Al4Ti4}=488\;MPa$). A higher strengthening effect was observed on the Al5Ti5 and Al6Ti6 alloys where $\sigma_{Y,\;Al5Ti5}=669\;MPa$ and $\sigma_{Y,\;Al6Ti6}=1190\;MPa$. Alloys containing Ti also showed greater deformation strength than alloys without Ti, where the maximum stress $\sigma_{max}$ after 50% deformation was found in alloys Al2Ti2, Al3Ti3, and Al4Ti4 at 1760 MPa, 2010 MPa, and 2129 MPa, respectively. In alloys Al5Ti5 and Al6Ti6, fracture occurred at a deformation of 43% and 26%, where $\sigma_{max}$ is 2239 MPa and 1942 MPa, respectively. Plastic deformation also caused a significant increase in the hardness of individual structural phases, which is shown in Figure 8.

## 4. Discussion

### 4.1. Effect of Al and Ti Content on Microstructural Evolution

**Figure 11:**
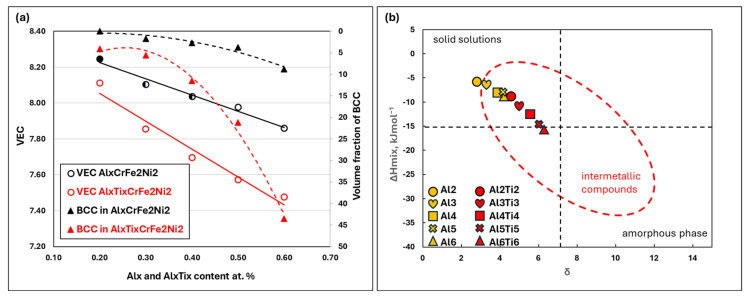
Graphical interpretation of the thermodynamic parameters in the as-cast alloys under study: (**a**) The relationship between VEC and the volume fraction of the BCC phase based on the content of Al_x_ and Al_x_-Ti_x_. (**b**) A prediction map of the studied alloys based on the extraction between ΔH_mix_ and δ.

HEAs can contain multiple phases, with the transformation or precipitation of the BCC phase from the parent FCC phase being influenced by chemical composition and thermodynamic conditions. In the medium- and high-entropy alloys, Al is often added to enhance strength properties, as it strongly stabilizes the BCC phase and promotes its precipitation from the FCC phase [25,35,36]. This was also confirmed in our Al_x_CrFe_2_Ni_2_ alloy (see Figure 11, black triangles), which shows the relationship between the Al content and the volume fraction of the ID region. Another element that effectively stabilizes the BCC phase is Ti [35,37,38]. Its positive effect on BCC phase formation, in synergy with Al, was demonstrated in our Al_x_Ti_x_CrFe_2_Ni_2_ alloy (see Figure 11, red triangles). Thermodynamic parameters for the characterization of HEAs, which are often used to predict structural stability and phase formation, are given in Table 9 for the studied alloys. The following empirical rules were used to calculate the factors representing discrepancies in atomic sizes (*δ*), the concentration of valence electrons (*VECs*), the enthalpy of mixing (Δ*H_mix_*), and a parameter *Ω*, which relates to the enthalpy of mixing, the entropy of mixing (Δ*S_mix_*), and melting temperature (*T_m_*) [13].

Based on the composition and configurational entropy provided in Table 9, only those alloys that meet at least one of the two main definitions of high-entropy alloys (HEAs) [39] could be classified as HEAs. These definitions are given as follows: (1) they contain at least five principal elements (*n_major_* ≥ 5), with each element having an atomic percentage between 5% and 35% (5% ≤ Xi ≤ 35%), or (2) they have configurational entropies in a random state greater than 1.5 R, regardless of whether they are single-phase or multiphase at room temperature (Δ*S_conf_* ≥ 1.5 R). Based on these definitions, the alloys Al4Ti4, Al5Ti5, and Al6Ti6 were considered HEAs because they met the compositional criteria and were close to the configurational entropy threshold. All other alloys were classified as medium-entropy alloys (MEAs).

The valence electron concentration (VEC), as proposed by Guo et al. [40,41], states that FCC phases are stable when VEC > 8, whereas FCC and BCC phases coexist in alloys with VEC in the range of 6.87 to 8. The VEC values for the studied alloys are plotted in Figure 11, where the trend lines show that the decrease in VEC is more pronounced in the Al_x_Ti_x_CrFe_2_Ni_2_ alloys compared to the Al_x_CrFe_2_Ni_2_ alloys. A single-phase FCC structure was observed only in the Al2 alloy with VEC > 8.1 (the fully closed black symbol is presented in Figure 11). The formation of a secondary phase in the form of ID nuclei was observed using SEM in the Al3 and Al4 alloys at 8.1 ≥ VEC ≥ 8 (the top-half closed black symbol in Figure 11), although it was not detected by XRD analysis. The same conclusion regarding this type of alloy was also reached by the authors of [25]. A clear presence of ID regions excluded from the FCC phase occurred when 8 ≥ VEC > 7.86 for the Al5 and Al6 alloys (the empty black symbol in Figure 11), as confirmed by XRD analysis. For the Al_x_Ti_x_CrFe_2_Ni_2_ alloys, the strengthening effect of Ti on the formation of the BCC phase was evident, with this effect already observed in the Al2Ti2 alloy at VEC = 8.1 (Figure 11). As the Al and Ti content in the alloy increased further, a sharp rise in the BCC phase volume fraction was observed, reaching 44% when VEC decreased to 7.86.

One of the key parameters influencing the formation of solid solutions in HEAs is the atomic size difference, δ. This parameter directly affects the FCC lattice parameter, thereby increasing the instability of the solid-solution phase. The studied alloys were designed to achieve a gradual increase in the δ parameter (see Table 9 and Figure 11b). Several authors [42,43] have proposed a criterion to predict the stability range of saturated solid solutions based on δ, which should lie within the interval 1% < δ < 6.6%, and mixing enthalpy ΔH_mix_ within the range of −15 kJ∙mol^−1^ ≤ ΔH_mix_ ≤ 5 kJ∙mol^−1^. The graphical interpretation of this criterion, according to Guo et al., for the studied alloys is shown in Figure 11. Based on the thermodynamic parameters (2.89% < δ < 6.25% and −5.8 kJ∙mol^−1^ ≤ ΔH_mix_ ≤ 15.3 kJ∙mol^−1^), it can be concluded that the phase prediction using the aforementioned criterion, as well as VEC, is in good agreement with the phases observed in this study.

### 4.2. The Relationship Between Structure and Deformation Behavior

**Figure 12:**
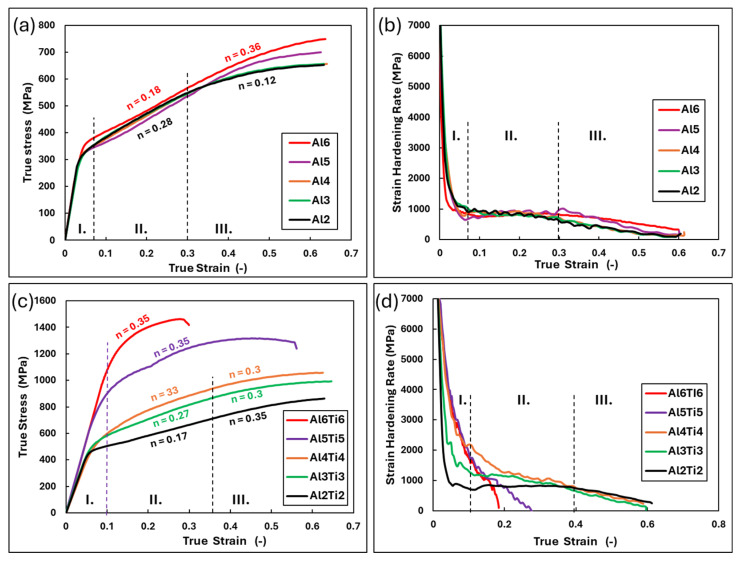
Mechanical properties of the analyzed alloys expressed as true stress–strain curves for Al_x_CrFe_2_Ni_2_ (**a**) and Al_x_Ti_x_CrFe_2_Ni_2_ (**c**); the strain-hardening rates as a function of true strain for Al_x_CrFe_2_Ni_2_ (**b**) and Al_x_Ti_x_CrFe_2_Ni_2_ (**d**).

When evaluating the plastic properties of the materials in detail, it is necessary to rely on the true stress values, which account for changes in the sample geometry during deformation. These values provide accurate information about the material’s behavior during plastic deformation in processing technologies [35]. The true stress–strain curves, along with the strain-hardening rate plots of all studied alloys, are presented in Figure 12. The strain-hardening exponent n was estimated for each curve. The Al_x_CrFe_2_Ni_2_ alloys, due to their single-phase FCC structure achieved through homogenization annealing, exhibited excellent ductility. In particular, the Al2, Al3, and Al4 alloys, whose curves completely overlap, demonstrated outstanding ductility. After entering the plastic state, this ductility was characterized by uniform deformation at a constant strain-hardening value of n = 0.28, which decreased to n = 0.12 at strains φ > 0.3. These two stages of plastic flow were also confirmed by the strain-hardening rate curve, where the hardening rate remained constant in Stage II, followed by a slight decrease in Stage III. A slight effect of increased Al content on the deformation behavior of the solid solution was observed in the Al5 and Al6 alloys. In these alloys, the uniform deformation in Stage II was accompanied by a minimal strain-hardening value of n = 0.18, which doubled to n = 0.36 in Stage III. These alloys exhibited better deformation properties than annealed stainless steel 316 L/LN at room temperature (where $\sigma_{Y,\;316LN}=325\;MPa$, $\sigma_{f,\;316LN}=641\;MPa,$ and $\varepsilon_{f,\;Al6Ti6}=49\;\%$) [15,44].

The single-phase Al2Ti2 alloy demonstrates similar deformation behavior and is comparable to alloys such as (FeCoNi)_94_Ti_6_ and Ni_46_Cr_23_Co_23_Al_4_Ti_4_ [45,46]. Further increases in the Ti content cause a gradual decrease in the strain-hardening rate during the initial stages of deformation (Region I in Figure 12), with a proportional decline in Region II for the Al5Ti5 and Al6Ti6 alloys until material cohesion fails. The highest true stress was exhibited by the Al6Ti6 alloy, with $\sigma_{max}^{true}=1437\;MPa$ at ε = 15.7%. This alloy demonstrates superior mechanical properties compared to the multicomponent alloy AlCrFe_2_Ni_2_ [47].

**Figure 13:**
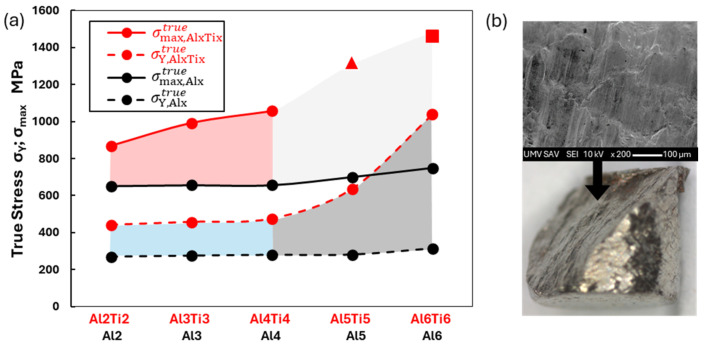
(**a**) Influence of chemical composition on the development of mechanical properties of the investigated alloys; (**b**) details of fracture in Al5Ti5 alloy.

A comparative analysis of the deformation properties of both studied alloy groups is shown in Figure 13. From the graphical dependence, it can be observed that during the initial stages of deformation ($\sigma_{Y}^{true}$), Ti exhibits a uniform strengthening effect in the Al2Ti2, Al3Ti3, and Al4Ti4 alloys compared to the alloys without Ti content, where $\sigma_{Y,AlxTix}^{true}=1.6x\sigma_{Y,Alx}^{true}$ (blue region). With further increases in the Ti content (alloys Al5Ti5 and Al_6_Ti_6_), there is a sharp rise in $\sigma_{Y,AlxTix}^{true}$ (gray region). At advanced deformation stages (φ = 0.65), it can be observed that the increasing Ti content enhances strengthening (red region). For alloys Al_5_Ti_5_ and Al_6_Ti_6_, this led to the exhaustion of plasticity, resulting in premature cohesion failure due to transcrystalline brittle fracture. A detailed view of the fracture is shown in Figure 13b. The strain-hardening potential, calculated as $\sigma_{max}^{true}/\sigma_{Y}^{true}$, evaluates the maximum achievable hardening of the material during plastic deformation before failure. According to [48], all studied alloys are classified as highly strain-hardening materials, where $\sigma_{max}^{true}/\sigma_{Y}^{true}\geq 1.9$.

## 5. Conclusions

In this work, the evolution of microstructures and mechanical properties of Co-free medium-to-high-entropy alloys based on a non-equimolar Al_x_Ti_x_CrFe_2_Ni_2_ system in the as-cast and annealed states was systematically investigated by SEM, XRD, and hardness characterization. The effect of the Al and Ti content on microstructures and pressure properties was analyzed and discussed. The main conclusions are given as follows:

- Phase prediction based on thermodynamic calculations such as VEC, δ, ΔH_mix_, and others were in good agreement with the obtained structures for all studied alloys in the as-cast state.
- The medium-entropy alloys Al_x_CrFe_2_Ni_2_ have a single-phase FCC structure in the as-cast state only up to an Al content of ≥3.85 at. %. As the Al content increases to 10.7 at. %, the structure transitions to a dual-phase FCC + BCC, with the volume fraction of BCC increasing to 8.8%. After homogenization annealing, all alloys reached a single-phase FCC structure, which is characterized by good ductility and a moderate-to-high strain-hardening exponent (n < 0.12; 0.36>). The Al_0.6_CrFe_2_Ni_2_ alloy, after 50% deformation, exhibited the highest compressive strength, $\sigma_{max}=1511\;MPa$, and a hardness of 359 HV0.1.
- Alloys of Al_x_Ti_x_CrFe_2_Ni_2_, alloyed with equal ratios of Al and Ti, exhibited a dendritic structure composed of FCC and BCC phases in the as-cast state across all concentrations. After homogenization annealing, only the Al_2_Ti_2_CrFe_2_Ni_2_ alloy exhibited a single-phase FCC structure. For the other alloys, as the Al and Ti content increased, the volume fraction of the BCC phase in the structure increased up to 61% for the Al_0.6_Ti_0.6_CrFe_2_Ni_2_ alloy. The highest compressive strength, $\sigma_{max}=2239\;MPa$, was achieved by the Al_0.5_Ti_0.5_CrFe_2_Ni_2_ alloy at 43% deformation. Premature failure of the alloy with the highest Al and Ti content (Al_0.6_Ti_0.6_CrFe_2_Ni_2_) was caused by a significant decrease in the strain hardening rate, which occurred at 26% deformation and $\sigma_{max}=1942\;MPa$.

## Figures and Tables

**Table 1:** Lattice constants of Al_x_CrFe_2_Ni_2_ alloys.

Microstructure		Lattice Constant (Å)				
	Samples	Al2	Al3	Al4	Al5	Al6
as-cast	FCC	3.592	3.603	3.593	3.607	3.599
	BCC	-	-	-	3.333	2.877
as-annealed	FCC	3.588	3.600	3.593	3.602	3.600

**Table 2:** Chemical compositions of different regions in as-cast Al_x_CrFe_2_Ni_2_ alloys obtained from EDS (at. %).

Alloys	Region	Chemical Composition (at. %)			
		Al	Cr	Fe	Ni
Al_0.2_CrFe_2_Ni_2_	Nominal	3.85	19.23	38.46	38.46
	Actual	3.52	18.39	38.53	39.57
Al_0_._3_CrFe_2_Ni_2_	Nominal	5.66	18.87	37.74	37.74
	Actual	5.00	19.09	39.12	36.79
	Dendrite	4.60	18.23	39.64	37.52
	Inter-dendrite	18.18	5.57	12.17	45.25
Al_0_._4_CrFe_2_Ni_2_	Nominal	7.41	18.52	37.04	37.04
	Actual	7.54	17.72	36.33	38.42
	Dendrite	5.75	17.04	38.72	38.50
	Inter-dendrite	18.12	10.10	26.88	44.90
Al_0_._5_CrFe_2_Ni_2_	Nominal	9.09	18.18	36.36	36.36
	Actual	8.29	17.77	36.63	37.31
	Dendrite	7.76	18.10	36.54	37.60
	Inter-dendrite	17.21	8.51	22.17	52.11
Al_0_._6_CrFe_2_Ni_2_	Nominal	10.71	17.86	35.71	35.71
	Actual	10.13	17.80	35.93	36.14
	Dendrite	8.79	17.33	38.03	35.86
	Inter-dendrite	24.18	11.34	17.34	47.14

**Table 3:** Chemical compositions of different phases/regions of as-annealed Al_x_CrFe_2_Ni_2_ alloys obtained from EDS (at. %).

Alloys	Region	Chemical Composition (at. %)			
		Al	Cr	Fe	Ni
Al_0.2_CrFe_2_Ni_2_	Actual	3.41	18.56	37.77	40.26
Al_0.3_CrFe_2_Ni_2_	Actual	4.86	18.98	36.78	39.38
Al_0.4_CrFe_2_Ni_2_	Actual	6.94	17.77	36.36	38.94
Al_0.5_CrFe_2_Ni_2_	Actual	8.00	18.52	35.09	38.39
Al_0.6_CrFe_2_Ni_2_	Actual	10.00	17.56	35.51	36.93

**Table 4:** Yield stress $\sigma_{Y}$ and maximal stress $\sigma_{max}$ at deformation ε=50% of the Al_x_CrFe_2_Ni_2_ alloys.

	Al2	Al3	Al4	Al5	Al6
$\sigma_{Y}\;(MPa)$	270	286	295	303	327
$\sigma_{max}\;(MPa)$	1302	1309	1316	1390	1511

**Table 5:** Lattice constants of Al_x_Ti_x_CrFe_2_Ni_2_ alloys.

Microstructure		Lattice Constant (Å)				
	Samples	Al2	Al3	Al4	Al5	Al6
as-cast	FCC	3.602	3.611	3.611	3.602	3.617
	BCC 1	-	3.385	2.932	2.923	2.887
	BCC 2	-	-	-	-	3.011
as-annealed	FCC	3.607	3.620	3.617	3.628	3.612
	BCC 1	-	-	2.927	2.935	2.867
	BCC 2	-	-	-	-	2.943

**Table 6:** Chemical compositions of different regions of as-cast Al_x_Ti_x_CrFe_2_Ni_2_ alloys obtained from EDS (at. %).

	Region	Al	Ti	Cr	Fe	Ni
Al_0.2_Ti_0.2_CrFe_2_Ni_2_	Nominal	3.70	3.70	18.52	37.04	37.04
	Actual	3.58	3.63	17.90	35.15	39.74
	Dendrite	3.15	1.93	17.64	37.17	40.10
	Inter-dendrite	2.63	13.86	8.94	17.97	56.59
Al_0.3_Ti_0.3_CrFe_2_Ni_2_	Nominal	5.36	5.36	17.86	35.71	35.71
	Actual	5.41	5.56	17.76	36.05	35.22
	Dendrite	5.87	5.08	18.49	34.76	35.81
	Inter-dendrite	18.18	18.83	5.57	12.17	45.25
Al_0.4_Ti_0.4_CrFe_2_Ni_2_	Nominal	6.90	6.90	17.24	34.48	34.48
	Actual	6.92	7.04	17.66	34.53	33.85
	Dendrite	6.75	6.76	18.07	34.77	33.66
	Inter-dendrite	18.92	18.13	5.04	13.25	44.65
Al_0.5_Ti_0.5_CrFe_2_Ni_2_	Nominal	8.33	8.33	16.67	33.33	33.33
	Actual	8.28	8.89	16.53	32.66	33.63
	Dendrite	4.80	8.44	18.85	35.91	32.00
	Inter-dendrite	19.23	19.79	4.04	12.70	44.23
Al_0.6_Ti_0.6_CrFe_2_Ni_2_	Nominal	9.68	9.68	16.13	32.26	32.26
	Actual	10.03	9.72	16.14	29.62	34.51
	Dendrite	5.44	5.69	17.73	37.54	33.59
	Inter-dendrite	17.62	13.95	6.12	12.80	49.50

**Table 7:** Chemical compositions of different regions of as-annealed Al_x_Ti_x_CrFe_2_Ni_2_ alloys obtained from EDS (at. %).

	Region	Al	Ti	Cr	Fe	Ni
Al_0.2_Ti_0.2_CrFe_2_Ni_2_	Matrix	3.58	3.63	17.90	35.15	39.74
	Black particle	9.37	87.71	1.23	1.69	-
Al_0.3_Ti_0.3_CrFe_2_Ni_2_	Matrix	4.89	5.18	17.66	36.16	36.10
Al_0.4_Ti_0.4_CrFe_2_Ni_2_	Matrix	6.02	4.25	18.59	36.80	34.35
	Phase	8.11	13.92	12.59	24.65	40.75
Al_0.5_Ti_0.5_CrFe_2_Ni_2_	Matrix	6.49	4.19	18.27	37.40	33.67
	Phase	5.51	19.67	9.74	19.98	44.80
Al_0.6_Ti_0.6_CrFe_2_Ni_2_	Matrix	6.40	4.78	17.74	38.05	33.03
	Phase	15.36	9.93	9.32	21.95	43.44

**Table 8:** Yield strength and maximum peak strength by 50% plastic strain of the Al_x_Ti_x_CrFe_2_Ni_2_ alloys.

	Al2Ti2	Al3Ti3	Al4Ti4	Al5Ti5	Al6Ti6
$\sigma_{Y}$	447	475	488	669	1190
$\sigma_{max}$	1760	2010	2129	2239	1942

**Table 9:** The thermodynamic parameters, VEC, ΔH_inf_, and ΔH_f_, of the investigated alloys.

Alloys	δ %	ΔHmix, kJ mol^−1^	ΔSmix, J mol^−1^ K^−1^	Ω, -	VEC -	Tm °C
Al_2_	2.77	−5.62	1.16 R	3.10	8.25	1592
Al_3_	3.27	−6.28	1.20 R	2.86	8.10	1586
Al_4_	3.94	−7.57	1.24 R	2.40	8.04	1554
Al_5_	4.11	−7.86	1.25 R	2.33	7.98	1548
Al_6_	4.48	−8.64	1.27 R	2.14	7.86	1533
Al_2_Ti_2_	4.18	−8.65	1.28 R	2.23	8.11	1593
Al_3_Ti_3_	5.02	−10.68	1.36 R	1.90	7.86	1583
Al_4_Ti_4_	5.54	−12.38	1.41 R	1.69	7.70	1572
Al_5_Ti_5_	6.00	−14.30	1.45 R	1.50	7.57	1555
Al_6_Ti_6_	6.28	−15.83	1.48 R	1.36	7.48	1538

## Data Availability

The data are contained within the article.
